# Autologous fat grafting for breast reconstruction after breast cancer: a 12-year experience

**DOI:** 10.1007/s00404-021-06241-1

**Published:** 2021-09-16

**Authors:** Sally Kempa, Eva Brix, Norbert Heine, Vanessa Hösl, Catharina Strauss, Andreas Eigenberger, Vanessa Brébant, Stephan Seitz, Lukas Prantl

**Affiliations:** 1 Department of Plastic, Aesthetic and Reconstructive Surgery, University of Regensburg, Franz-Josef-Strauss-Allee 11, D-93053 Regensburg, Germany; 2 Department of Obstetrics and Gynecology, Caritas Hospital St. Josef, University of Regensburg, D-93053 Regensburg, Germany

**Keywords:** Autologous fat grafting, Transplantation, Breast Cancer, Reconstruction, Safety

## Abstract

**Purpose:**

The aim of our study was to examine the surgical outcome and complications (efficiency) as well as the incidence of locoregional recurrence and distant metastases (oncological safety) in patients who underwent autologous fat grafting (AFG) of the breast following breast cancer surgery.

**Methods:**

In our monocentric cohort study, retrospective and prospective data were collected from all consecutive patients who underwent AFG after breast cancer between 2008 and 2020; a total of 93 patients met the inclusion criteria.

**Results:**

Our long-term results showed no increase in tumor recurrence and distant metastases in the studied collective when compared to the available literature. We observed 1 local recurrence (1.1%), 2 distant metastases (2.2%), and 1 tumor-related death (1.1%). There was a high degree of patient satisfaction; 67.12% of patients reported adequate satisfaction with autologous fat grafting.

**Conclusion:**

Currently, to our knowledge, this is the study with the longest follow-up time (mean 6.7 years after AFG and 11.5 years after tumor resection). The results of our clinical study will contribute to improve evidence in the broad field of AFG, adipose stem cell and tumor research. Consistent with our study, the literature review shows a clear tendency of clinical trial results with a low incidence rate of tumor recurrence and metastasis following the use of AFG. AFG seems to be a safe procedure also after breast cancer treatment.

## Introduction

Based on current incidence rates, breast cancer is the most commonly occurring cancer in women and the second-most common cancer overall [1]. Treatment often involves surgery, including breast-conserving surgery (BCS) or mastectomy. This is often combined with radiotherapy, chemotherapy, hormonal therapy, or a combination of all three.

However, despite numerous innovations, the reconstruction of the female breast still remains a huge challenge. Breast reconstruction and correction of contour defects of the breast by autologous fat grafting (AFG), having been performed for many years now, offer numerous advantages. These include the removal of autologous fat by liposuction from areas of (unwanted) fat accumulations, which has a much lower risk of complications and shorter operative times than performing larger flap surgeries. Reconstruction with implants is also a common type of breast reconstruction, however, many patients find the idea of using their own tissue more convincing than implanting a synthetic foreign body. Additionally, autologous fat is thought to have positive regenerative capabilities due to stem cells contained in the stromal vascular fraction (SVF). Unfortunately, this regenerative ability of stem cells also raises key concerns regarding the oncological safety of AFG after breast cancer. Another disadvantage is the variable survival rate of fat cells, leading to unpredictable outcomes and repeated procedures. Fat necrosis may present a challenge in breast cancer follow-up, by forming scar tissue, oil cysts or calcifications.

The aim of our study was to examine the surgical outcome and complications (efficiency) as well as the incidence of locoregional recurrence and distant metastases (oncological safety) in patients who underwent AFG of the breast following breast cancer surgery.

## Materials and methods

### Study population and goal

In our monocentric cohort study, retrospective and prospective data were collected from all consecutive patients who underwent AFG after breast cancer in the University Center for Plastic, Aesthetic, Hand, and Reconstructive Surgery (Caritas St. Josef Hospital in Regensburg, Germany) between 2008 and 2020. A total of 93 patients met the inclusion criteria. Patients with invasive and in situ carcinoma of the breast who underwent BCS or mastectomy were included, regardless of postoperative treatment (radiation, chemotherapy, or hormone therapy) and prior breast reconstruction. The total inclusion and exclusion criteria are listed in Table 1.

**Table 1:** Inclusion and exclusion criteria

Inclusion criteria	Exclusion criteria
Female	Prophylactic mastectomy without cancer detection
Age 18 and above	Primary Metastases
Primary breast cancer	Soft-tissue Sarcoma (Cystosarcoma Phylloides, Pleomorphic Sarcoma)
Primary tumor resection	Inflammatory Breast Cancer
Regular follow-up	Secretory Breast Cancer
	Less than 6 months of follow-up time after AFG
	Tumor recurrence before AFG

Formal and documented ethical approval was obtained (reference number 18-1226-101), and to ensure optimal quality of data reporting, the STROBE (Strengthening the Reporting of Observational Studies in Epidemiology) guidelines were followed when designing and reporting the study.

The primary endpoint of this study was the incidence of locoregional tumor recurrence or distant metastases, and time from tumor surgery to the oncologic event. Secondary endpoints were subjective graft survival, patient satisfaction, and number of complications and/or required biopsies.

### Surgical technique

The surgeries were performed under general anesthesia. Liposuction and AFG were performed applying the Coleman technique or water-jet assisted (WAL) without further stem cell enrichment. Preferred harvest sites were abdominal subcutaneous adipose tissue or adipose tissue from the thighs. In some patients, a vacuum-based external breast expander (BRAVA® system (LLC Miami, FL, USA)) was applied both pre- as well as postoperatively [2]. Previous breast reconstruction techniques included tissue expander insertion or implant reconstructions, oncoplastic reconstructions, and flap reconstruction. No AFG was performed as an immediate breast reconstruction.

### Data collection

First, clinical or pathological data of patients treated with AFG were systematically collected retrospectively using the hospital's internal documentation system (MCC Meierhofer®, Meierhofer AG, Munich, Germany).

The following data were taken from patient files: patient age at tumor surgery, body mass index (BMI), date and type of tumor resection (mastectomy or BCS), tumor histology (in situ or invasive carcinoma), tumor classification (TNM stage; in case of multiple simultaneous tumors, the tumor with the highest T category was classified), grading, estrogen and progesterone receptor expression, Her2/neu receptor status, adjuvant chemotherapy, radiotherapy, or antihormone therapy, previous reconstruction type (no reconstruction, oncoplastic reconstruction, flap surgery, and/or implant or expander implantation), number/dates of AFG therapy sessions, total transplanted fat volume and existing risk factors (smoker, diabetes, anticoagulation) at the time of the first AFG session.

After acquisition of data sets, a structured telephone follow-up was then performed and the following variables were obtained: complications after AFG (fat necrosis/oil cyst, contour irregularity, infection), subjective graft retention rate (0–100%), aesthetic outcome for contour defects and volume asymmetries (100% = excellent, 75% = very good, 50% = good, 25% = poor, 0% = insufficient), oncologic follow-up (regularly, irregularly), number of necessary biopsies of the affected breast, occurrence of locoregional recurrences or metastases. To ensure that no oncologic events were missed, if a patient was not available by telephone (*n* = 8), survival status was determined from the Regensburg Breast Cancer Registry, or by inquiring at the residents' registration office. Survival status could not be determined for three patients; therefore, they were not included in the analysis (*n* = 90). If no events occurred, the study endpoint was censored at the last follow-up. The end of observation for living patients without oncologic events or death during the study period was December 15, 2020.

## Results

### Study population

A total of 90 patients who underwent AFG after UICC stage 0 to IIIC breast cancer between 2008 and 2020 in our University Center for Plastic, Aesthetic, Hand and Reconstructive Surgery (Caritas St. Josef Hospital in Regensburg, Germany) were identified with complete clinicopathological data. The characteristics of the study population are shown in Table 2. The mean age at breast cancer surgery was 46.1 (21–69) years and of the 90 patients with complete tumor stages, 13 patients had in situ carcinomas and 77 had invasive carcinomas. 51.1% of patients had favorable tumor stages (UICC stage 0 or I). 78.1% of patients had estrogen receptor (ER)-positive disease. On average, two AFG sessions were performed per patient (range: one to seven sessions).

**Table 2:** Description of the study population

Variable	Classification	Value (*n* = 90)
Mean age at breast cancer surgery (SD) in years		46.1 (9.6)
Mean-BMI (SD) in kg/m^2^		24.1 (3.5)
Tumor surgery	BCS Mastectomy	20 70
Histology	In situ Invasive	13 77
UICC Stadium^*a*^	0 IA and IB IIA and IIB IIIA, IIIB and IIIC	13 33 24 20
Her-2-Status^a^	Positive Negative	21 46
Estrogen receptors^*a*^	ER+ PR+ ER+ PR− ER− PR−	59 9 19
Adjuvant therapy	Chemotherapy Radiotherapy Radio-chemotherapy None	17 12 39 22
Antihormone therapy	Yes No	63 27
Breast reconstruction	No reconstruction Flap surgery Oncoplastic reconstruction Implant/ Tissue expander Flap surgery and implant	28 32 4 22 4
Mean number of AFG sessions (SD)		2 (1.4)
Mean total transplanted fat volume (SD), ml		407 (444)
Risk factors	None Smoker Diabetes Anticoagulation	77 8 4 1

*BMI* body mass index, *SD* standard deviation

^a^Information not available for all patients

### Oncological events

The mean total follow-up time in our study was 11.5 (1.9–31) years after primary tumor surgery and 6.7 (0.6–11.6) years from first fat grafting to last follow-up. The mean time interval from breast cancer surgery to first AFG intervention was 4.8 (0.3–22.3) years. During this time, we observed 1 local recurrence (1.1%), 2 distant metastases (2.2%), and 1 tumor-related death (1.1%). No locoregional recurrences were observed. The time interval between tumor surgery and follow-up at which recurrence was first diagnosed (tumor-free interval) was 1.2 years after the first AFG session and 2.7 years after tumor resection (Table 3).

**Table 3:** Locoregional recurrence and distant breast cancer metastasis

Age at tumor surgery (years)	Histology and primary tumor localization	UICC-Stadium	Treatment	Total fat vol. (ml)	Time interval*		Oncologic event
					AB	BC	
48	T1c N1a M0, G2 ER+ PR– Her2− Right upper medial quadrant	IIA	BCS, RT, Tamoxifen	200	1.5	1.2	Local recurrence: T1c N0 M0, G3, ER− PR− Her2neu+ Right upper medial quadrant
29	T1 N0 M0, G3 ER+ PR+ Her2−	IA	Mastectomy, CT, Tamoxifen	120	3.9	7.1	Lung metastasis
42	T1b N0 M0, G2 ER+ PR− Her2+	IA	Mastectomy, CT, Tamoxifen	680	2.2	0.6	Liver metastasis
47	T3m N1a M0, G2 ER− PR− Her2+	IIIA	Mastectomy	50	10.8	4.7	Tumor-related death

*Time intervals in years: AB (tumor surgery to AFG), BC (AFG to end of follow-up/locoregional recurrence/metastasis or tumor-related death), BCS (breast-conserving tumor resection), RCT (radio-chemotherapy), CT (chemotherapy)

**Fig. 1:**
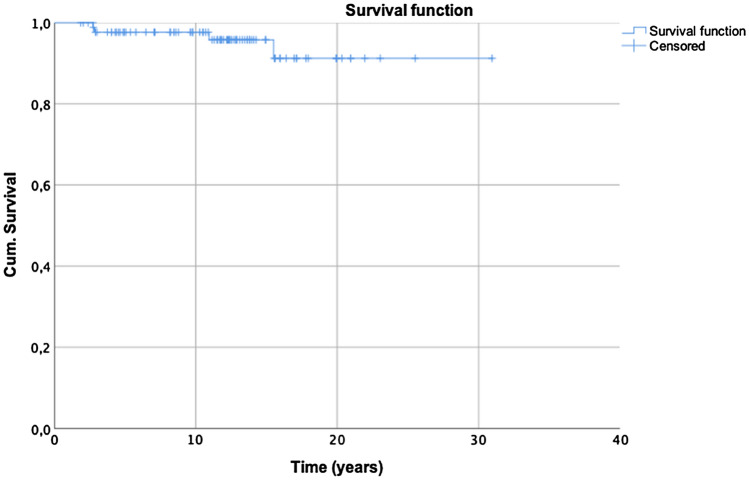
Kaplan–Meier curve of recurrence-free interval

The disease-free survival (tumor recurrence or metastasis) is 97.7% after 10 years and 91.2% after 30 years (Fig. 1).

### Secondary endpoints

Overall, the biopsy rate was 16.7% (11.1% in the cohort after mastectomy and 5.6% after breast-conserving tumor resection). There was a high degree of patient satisfaction; 67.12% of patients reported they were satisfied with autologous fat grafting (excellent, very good, good). The estimated average healing rate was 52.5%. Oil cysts and fat necrosis were the most frequently reported complications (17.0%), while 4% of patients had contour deformities at the liposuction areas, and 2% of patients had an infection in the recipient area that required antibiotic treatment.

## Discussion

Despite early detection and guideline-based treatment of breast cancer, a proportion of patients develop tumor recurrence or metastases months to years later [3]. There are different prognostic factors that, individually or in combination, may favor tumor recurrence. Breast cancer recurrence is a multifactorial phenomenon: tumor size, estrogen receptor status, Her2/Neu expression, Ki-67 proliferation rate, and young age all increase the risk of local recurrence [4]. In the search for causes, numerous theories explaining its occurrence can be found; for most tumor recurrences, it is believed that there is an association with the reactivation of dormant disseminated tumor cells (DTCs), that once the microenvironmental conditions are favorable, transition into proliferating cells and induce tumor progression [5]. The interactions are complex and our understanding of these processes is still vastly limited [6].

Not only tumor growth, but any surgical procedure, (i.e. tumor surgery itself or secondary breast reconstruction regardless of the type of reconstruction procedure) induces local hypoxia and tissue trauma with subsequent wound healing [7]. Hypoxia-inducible factors (HIFs) mediate adaptive physiological responses to hypoxia; HIF activity in regions of intra-tumoral hypoxia mediates angiogenesis, epithelial–mesenchymal transition, stem cell maintenance, invasion, metastasis, and resistance to radiation therapy and chemotherapy [8]. Studies of the dynamics of metastasis occurrence in patients with and without breast reconstruction (flap surgery, implant, or combined procedures) after breast cancer revealed a similar bimodal increase in tumor recurrence at 2 and 5–6 years when the time origin date is placed at mastectomy date and at reconstruction date [9]. One possible explanation is that the mechanical trauma of surgery temporarily induces a systemic inflammatory response and thus, via proinflammatory or angiogenic mediators, may affect apparently latent-state tumor cells [9].

The glandular tissue of the female breast is surrounded by adipose tissue. Adipocytes and their precursor cells can influence tumor behavior via various hormones, growth factors, and cytokines known as adipokines including leptin, adiponectin, IL-6, hepatocyte growth factor, autotaxin, and TNF-alpha [10–12] although their exact mechanism of action has not been established. A particular focus here lies on the adipose-derived stem cells (ASC), contained in transplanted fat, and their controversial yet crucial and complex dual role as tumor promoters and suppressors. Numerous experimental series exist in which mesenchymal stem cells are cultured together with different tumor entities in vitro or in vivo, with varying results [13, 14]. This contradiction might be due to the heterogeneity within stem cell subgroups and their extracellular vesicles, with polarization into antitumor stem cells and protumor stem cells [15, 16]. Furthermore, it should be noted that in contrast to the partially immunodeficient experimental animals receiving purified adipose-derived stem cell cultures, human immunocompetent patients typically receive adipose grafts with a variable but small proportion (2–8%) of adipose-derived stem cells [17]. The tumor stem cells themselves used in laboratory experiments are also highly tumorigenic, even when inoculated in small numbers [18].

Despite the routine clinical use of autologous fat grafting, numerous clinical studies have failed to establish any association between autologous fat grafting and increased incidence of local or systemic breast cancer recurrence [19–22]. The only exception actually confirming a carcinogenic potential was found by Petit et al. [23, 24] in a subgroup of patients with ductal and lobular intraepithelial neoplasia. This could not be confirmed in our study, possibly due to the small number of cases (*n* = 13). Currently, to our knowledge, this is the study with the longest follow-up time (mean 6.7 years after AFG and 11.5 years after tumor resection). The long-term experience with AFG showed no increase in tumor recurrence and distant metastases in the studied collective when compared to the available literature. The incidence rate of local recurrence or metastasis after exposure to autologous fat was 0.6% per year. Krastev et al. [25] reported a cumulative incidence rate of 0.73% per year for the entire cohort in the largest meta-analysis of autologous fat transplantation performed to date (*n* = 4292), confirming our finding.

Limitations of our study included a retrospective setting and the lack of a control group. However, in the present study, where a causal effect is the parameter of interest, we deliberately used the unmatched design due to the expected low number of tumor recurrences and distant metastases. Currently, breast reconstruction with autologous fat is performed in many hospitals, but there is a lack of a central registry with an accurate protocol for documenting patient data (including molecular subtypes of breast cancer variants, BRCA status, and uniform demographic and procedure-specific data as well as defined follow-up dates) also in collaboration with breast cancer registries.

With regard to the secondary endpoints of our study, the overall satisfaction with the treatment was shown to be very high. Especially convincing is the easy extraction of autologous fat from body regions with (unwanted) fat accumulation through smallest incisions and the autologous character of the treatment method. In 17% of patients, fat tissue necrosis or oil cysts were detected post-operatively without the need for biopsies or revision surgery. The progressive improvement and optimization, among others of the preparation technique by the Cell Enriched Lipotransfer (CELT) method, will further improve the complication and healing rates with a higher engraftment (over 90%) of the lipoaspirate in the future [26–28].

## Conclusion

The results of our clinical study will contribute to improvements in the broad field of stem cell and tumor research. Consistent with our study, the literature review shows a clear tenor of clinical trial results with a low incidence rate of tumor recurrence and metastasis following the use of AFG after breast cancer. This contrasts with the equivocal preclinical studies. However, an increasing understanding of the factors and cell types involved, whose mechanisms of action have not yet been fully elucidated, is emerging here. Further multicenter prospective studies and a prospective clinical registry with high-volume multicenter data and a long follow-up period are needed to further demonstrate oncological safety.

## Data Availability

Available upon request.
